# Value of skin testing children with a family history of food allergy before ingestion of suspect food allergens

**DOI:** 10.1186/1710-1492-10-S2-A29

**Published:** 2014-12-18

**Authors:** Arunmozhi Dominic, Vaishaali Manga, Immaculate Nevis, Laura Kim, Harold Kim

**Affiliations:** 1 Division of Clinical Immunology and Allergy, Western University, London, Ontario, Canada; 2 Department of Clinical Epidemiology and Biostatistics, McMaster University, Hamilton, Ontario, Canada; 3 McGill University, Montreal, Quebec, Canada; 4 Division of Clinical Immunology and Allergy, McMaster University, Hamilton, Ontario, Canada

## Background

A family history of food allergy can cause anxiety in parents. This may prevent food introduction in their children. Current guidelines recommend skin testing only when there is a reaction to a food in that specific patient. When there is a family history of food allergy, parents frequently ask their physicians for food testing of their children prior to introduction of specific foods. We conducted this study to determine whether allergy skin testing reduces anxiety levels in parents thereby leading to food introduction.

## Methods

The parents of 50 children with a family history of food allergy completed a Visual Analog Score (VAS) to estimate their anxiety to give their children the specific food of concern. Previously, the children had not eaten the food. The VAS scores were recorded pre- and post-skin testing on a scale from 0 to 10. The likelihood of food introduction pre- and post-skin testing was estimated.

## Results

The mean age of the children was 3.6 years; the majority were males (62%). Approximately 58% of patients’ parents, 38% siblings and 4% other relatives had food allergy. Most children (78%) had family history of a single food allergen and 60% had a family history of allergy to peanuts. All children tested negative for the food allergen of concern. Mean VAS was statistically different pre- and post-skin testing (pre VAS mean= 7.83 vs post VAS mean = 2.15; p=<0.0001). The likelihood of food introduction pre- and post testing was 4% and 92% respectively.

## Conclusion

Skin testing reduces the anxiety of parents of children with a family history of food allergy prior to introduction of the food allergen of concern. The food is more likely to be introduced into the diet after negative skin testing. Although it did not occur in this study, there is a still a risk of false positive skin testing.

